# Feasibility of T1rho and T2 map magnetic resonance imaging for evaluating graft maturation after anatomic double-bundle anterior cruciate ligament reconstruction

**DOI:** 10.1186/s13018-019-1193-y

**Published:** 2019-05-16

**Authors:** Yasuo Niki, Takayuki Yasuoka, Shu Kobayashi, Kengo Harato, Takeo Nagura, Shigeo Okuda, Masahiro Jinzaki

**Affiliations:** 1 Department of Orthopaedic Surgery, School of Medicine, Keio University, 35 Shinanomachi, Shinjuku, Tokyo, 160-8582 Japan; 2 Department of Diagnostic Radiology, School of Medicine, Keio University, 35 Shinanomachi, Shinjuku, Tokyo, 160-8582 Japan

**Keywords:** Anterior cruciate ligament, Magnetic resonance imaging, T1rho mapping, T2 mapping, Anatomic double bundle reconstruction, Graft maturation

## Abstract

**Background:**

Although T1rho and T2 map magnetic resonance imaging (MRI) have been perceived as useful diagnostic modalities for cartilage degeneration, no studies have assessed whether these two sequences are useful for monitoring ACL graft maturation after ACL reconstruction. The present study examined whether the two sequences reflect graft function and maturation after ACL reconstruction.

**Methods:**

Twenty consecutive patients who consented to undergo MRI at 3, 6, and 12 months after double-bundle ACL reconstruction were enrolled. MRI was performed using T1 rho and T2 map sequences in a single session. Temporal changes in T1rho and T2 values of a purely tendinous portion of graft were assessed at each time point. Correlations were analyzed between T1rho or T2 map values and clinical results, including anteroposterior laxity at 2 and 4 years postoperatively, pivot shift test results at 4 years, and graft tension on second-look arthroscopy. Separate analyses were performed for the anteromedial bundle (AMB) and posterolateral bundle (PLB).

**Results:**

T1rho sequence was able to visualize the tendinous portions of AMB and PLB more clearly than T2 map sequence even on gray-scale images. Mean T1rho and T2 map values gradually decreased during the first operative year, but the trend was more prominent and consistent for T1rho values than for T2 map values. Correlation analysis revealed that T1rho and T2 map values at 1 year correlated significantly with anteroposterior laxity at 2 and 4 years. This trend was found in both AMB and PLB. Both T1rho and T2 map values failed to exhibit a statistical correlation with arthroscopic findings of graft tension.

**Conclusions:**

The present study was the first trial to assess the feasibility of T1rho and T2 map sequences to objectively monitor the course of graft maturation after ACL reconstruction. Both sequences successfully detected purely tendinous portions of graft, and mean values gradually decreased during the first year postoperatively. Both values at 1 year correlated significantly with anteroposterior laxity of the knee joint at 4 years, indicating that the values reflected graft fate.

## Introduction

Magnetic resonance imaging (MRI) is a noninvasive imaging modality that has been widely used to examine injuries to the anterior cruciate ligament (ACL). The diagnostic accuracy of MRI for ACL injuries is increasing and has already reached ≥ 90% [1]. However, a major concern has been raised regarding the difficulty of assessing reconstructed ACL grafts using MRI [2, 3]. Healthy ACL normally displays very low intensity on MRI, unless drastic changes such as rupture [4] or mucoid degeneration [5–7] occur. MRI with a contrast agent such as gadolinium diethylenetriamine pentaacetic acid has demonstrated not only neovascularization but also matrix synthesis along with graft maturation [8, 9]. In clinical practice, however, MRI findings fail to correlate with actual graft functions of the anterior and rotational stabilizers [3], which can be clinically evaluated using a KT-1000 arthrometer (Medmetric, San Diego, CA) and the pivot shift test, respectively.

T1rho and T2 map sequences have shown potential in musculoskeletal MRI at 3 T, and have been perceived as particularly useful diagnostic modalities for depicting early cartilage degeneration in osteoarthritic knees [10–13]. Although these two sequences have been shown to probe the water [14], collagen [15], and proteoglycan (PG) contents [16] of cartilage, no reports appear to have assessed the feasibility of using these two sequences to evaluate ACL graft maturation after ACL reconstruction. The present study examined the feasibility of using the two sequences to monitor the degree of graft maturation after ACL reconstruction. Moreover, temporal changes in signal intensity were compared between sequences. We hypothesized that graft signals from T1rho and T2 map sequences would correlate with anterior-posterior and rotational stability of the knee and that these would represent useful imaging modalities for probing graft function and maturation after ACL reconstruction.

## Materials and methods

### Subjects

Among 75 patients undergoing anatomic double-bundle ACL reconstruction using semitendinous tendon graft at our institute between January 2011 and January 2012, participants comprised 26 consecutive subjects who consented to participate in this study and undergo MRI at 3, 6, and 12 months postoperatively. All patients provided written informed consent, and an institutional review board approved the study (ID #2011266). Exclusion criteria were multiple ligament injuries (PCL or MCL), simultaneous bilateral ACL injuries, multiple operated knees, or inflammatory joint disease. According to this criterion, 26 patients were initial candidates eligible for analysis, but 6 patients were lost to follow-up, lost halfway, and did not complete three sequential MRIs. Finally, 20 patients were enrolled (Table 1).

**Table 1:** Demographic characteristics of patients

Number of patients	20
Age, years (range)	27.7 (15–45)
Sex (male/female)	6/14
BMI^a^	21.8 (18.2–23.8)
Time from injury to op. (months)^a^	26.7 (2–192)
Preinjury Tegner activity level^a^	6.4 (5–9)
Preoperative Lysholm score^a^	70.5 (41–82)
Surgical technique	Double-bundle reconstruction (graft: semitendinosus tendon)

^a^Data are expressed as mean (range)

### Clinical evaluation

Clinical assessment was performed at 1, 2, and 4 years postoperatively using knee stability tests including the manual pivot shift test and measurement of side-to-side differences in anteroposterior laxity with a KT-1000 arthrometer (Medmetric, San Diego, CA). Lysholm score was collected 4 years postoperatively. One orthopedic surgeon blinded to clinical scores and MRI findings performed all stability tests. Second-look arthroscopy was performed at the time of hardware extraction, at a mean of 18.9 months (range, 14–24 months) after ACL reconstruction. Grafts were evaluated for tension by probing at 20–90° of knee flexion, then classified into three categories as follows: tension same as normal ACL, taut; tension less than normal, mildly lax; and obvious loss of tension, lax.

### Imaging protocol

Assessment with MRI was performed at 3, 6, and 12 months postoperatively. All MR exams were implemented on a 3-T MR scanner (Discovery MR750; GE Healthcare Technologies, Waukesha, WI) equipped with an 8-channel transmit/receive knee phased array coil. In each sequence of T1rho and T2 map imaging, centers of the bone tunnel outlet for both femur and tibia were plotted on the axial plane, and the plane passing through these 2 points was the slice plane for oblique sagittal imaging. Single or double oblique imaging has been reported to contribute to an accurate diagnosis of ACL injury [17]. Centers of tunnel outlets were plotted for both femur and tibia, and the resultant axis passing through the 2 points indicated the putative graft axis. This graft axis was positioned at approximately 10–15° of internal rotation from the mechanical axis of the knee joint, when projected on the coronal plane. Based on this method, axes of the reconstructed anteromedial bundle (AMB) and posterolateral bundle (PLB) could be depicted separately.

### Measurement of graft signal intensity

MRI data were transferred to a workstation for offline quantification of T1rho and T2 map values. Three intra-articular locations of the graft at which slice planes could be made perpendicular to the long axis of the graft were defined on sagittal images. Those included the midpoint between femoral and tibial tunnel outlets and points 1 cm proximal and 1 cm distal to the midpoint. Segmentation was performed manually on the three slice planes to segregate the purely tendinous portion from surrounding synovial tissue. Regions of interests (ROIs) set on the purely tendinous portion of the graft were measured for relaxation times of T1rho and T2 map sequences, and the mean value of the three slice planes was calculated. Mean values for AMB and PLB were measured separately.

### Surgical techniques

All ACL reconstructions were performed arthroscopically by a single surgeon using anatomic double-bundle procedures, as reported previously [18, 19]. Briefly, semitendinosus tendon was harvested and cut into two pieces, and the two double-looped semitendinosus tendon grafts were prepared for AMB and PLB grafts. Arthroscopically, the ACL remnant was removed, and the bony ridge of the medial wall of the lateral femoral condyle (i.e., resident ridge) was exposed. Bone tunnels for AMB and PLB were placed posterior to the resident’s ridge, and centers of these tunnels corresponded to the center of each anatomic footprint, including the fan-like portion [20, 21]. Under arthroscopic observation through an anteromedial portal, the tip of a femoral outside-in ACL aimer set for insertion angles of 100° (Arthrex, Naples, FL) was placed precisely at the center of each footprint. A 3.5-mm guide pin was introduced from outside the joint through a small incision over the lateral femoral cortex. The guide pin was replaced by a FlipCutter® (Arthrex) 5–6 mm in diameter, which in turn cut a socket into the femur to a depth of 15 mm. Regarding creation of a tibial bone tunnel, two 2.4-mm guide pins were inserted into the center of the AMB and PLB footprints using a drill guide system (Arthrex). After 5.5–6.5 mm of overdrilling, grafts were introduced through the tibial tunnel into the femoral tunnel. The two semitendinosus tendon grafts were secured at the femur using TightRope® (Arthrex). For tibial graft fixation, two sets of small double spike plates (Smith & Nephew, Memphis, TN) were used. Grafts for both the AMB and PLB were independently secured with 20 N of tension with the knee in 20° of flexion using a ligament tensioner (Smith & Nephew).

### Statistical analysis

Spearman’s rank order correlation was performed to examine the relationship between T1rho or T2 map values at 1 year and anteroposterior laxity measured at 2 and 4 years postoperatively. Correlations of T1rho or T2 map values with pivot shift test results and graft tension at the time of second-look arthroscopy were also analyzed. The paired Student’s *t* test was used to statistically analyze differences among 3-month, 6-month, and 1-year T1rho and T2 map values. Values of *p* < 0.05 were considered statistically significant.

## Results

**Fig. 1:**
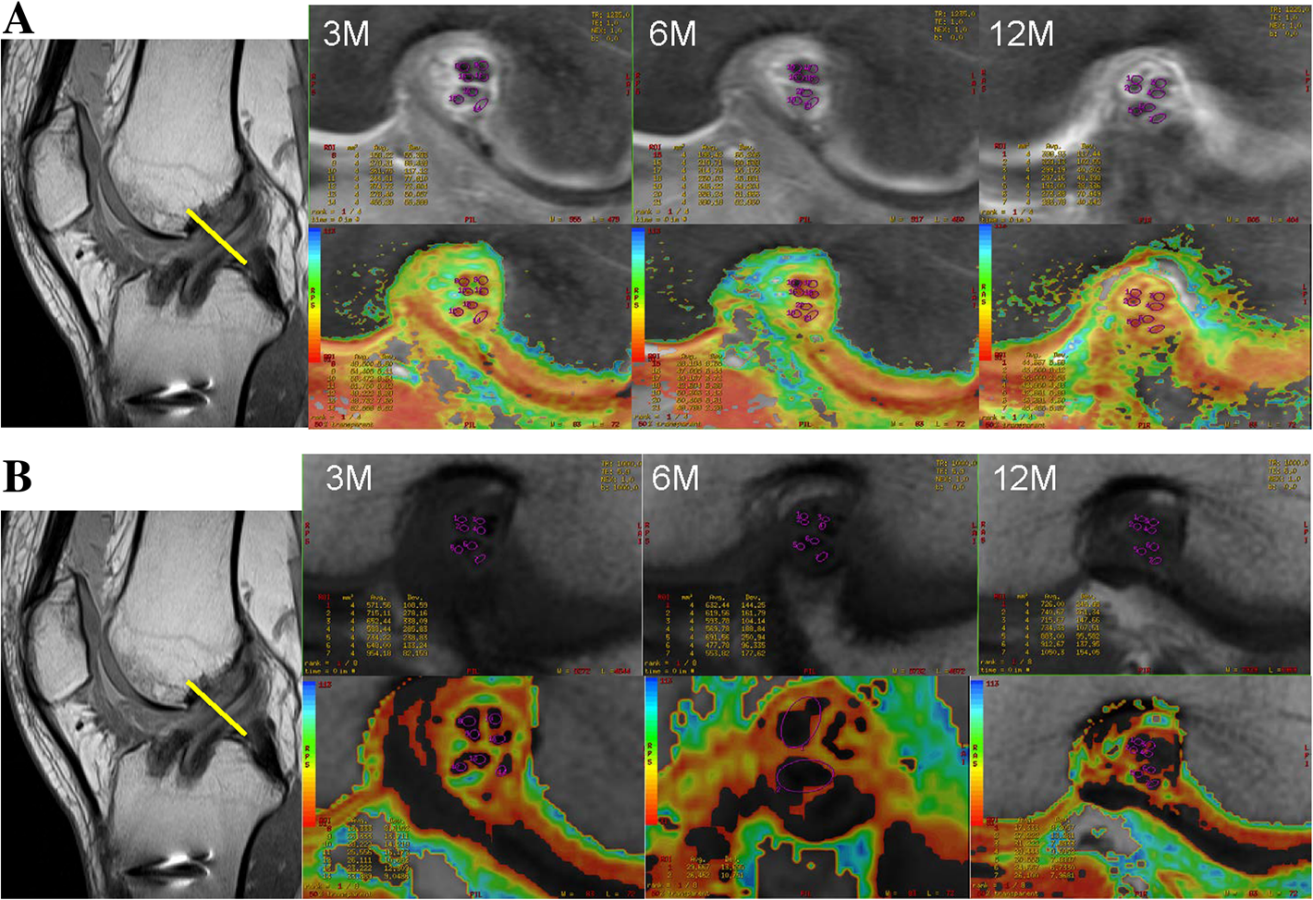
Representative images of the graft in the axial plane. Both gray-scale and color coding images for T1rho (**a**) and T2 map (**b**) at 3 months, 6 months, and 1 year are shown

**Fig. 2:**
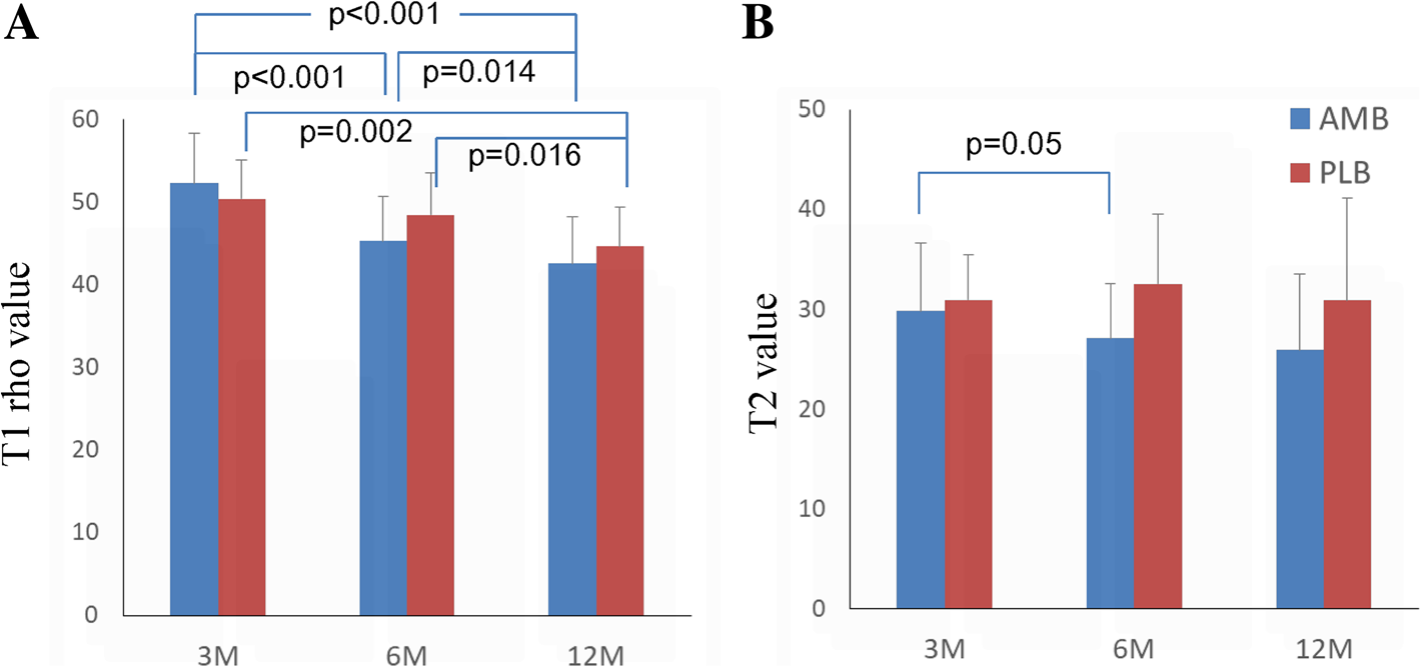
Temporal changes in mean T1 rho (**a**) and T2 values (**b**) during 1 year postoperatively

**Fig. 3:**
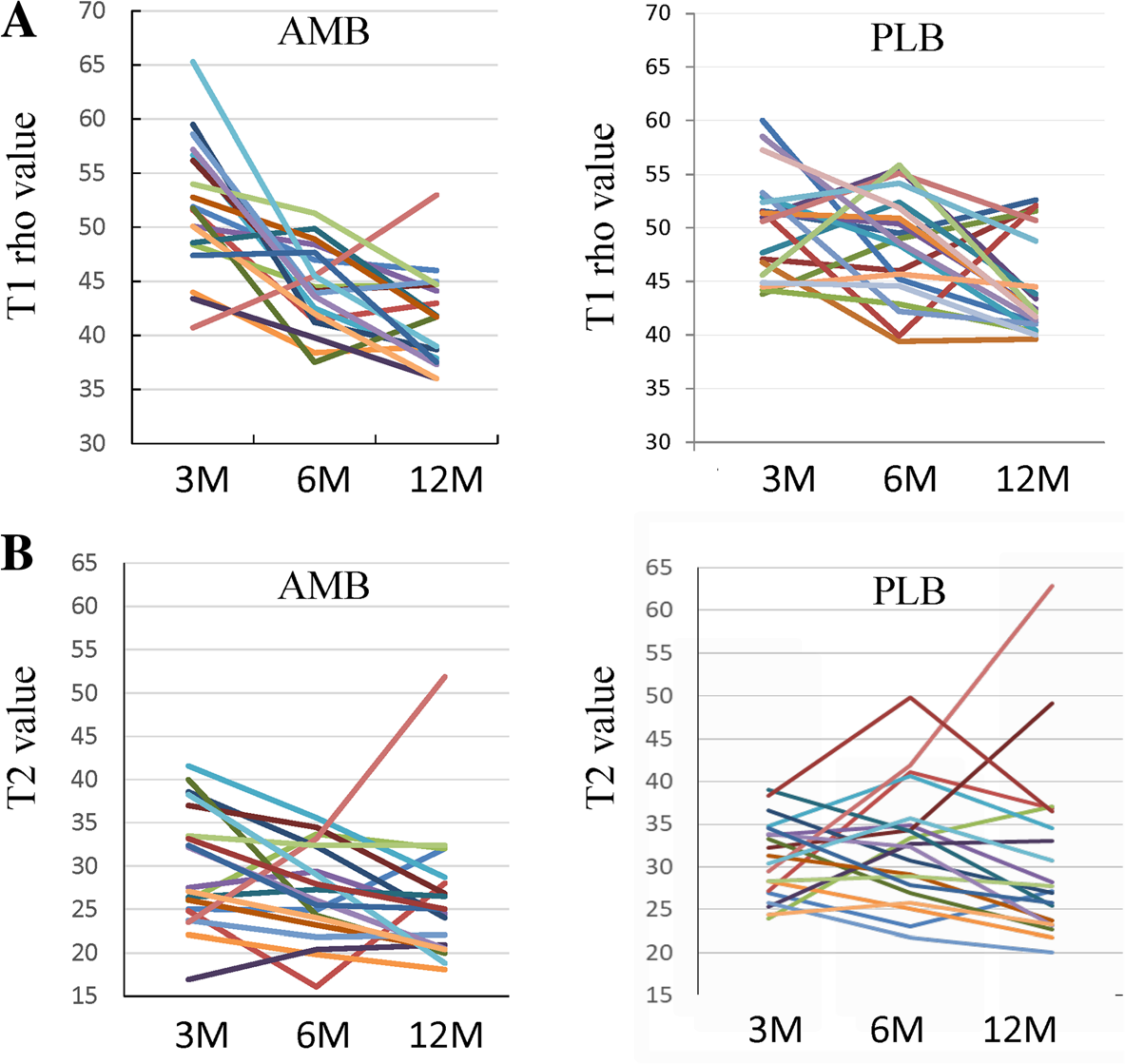
Temporal changes in individual T1 rho (**a**) and T2 values (**b**) during 1 year postoperatively

On MRI examination, the T1rho sequence was able to visualize tendinous portions of the AMB and PLB more clearly than the T2 map sequence in gray-scale images, and easily segregated the purely tendinous portion from surrounding synovial tissue (Fig. 1). Color-coded images were capable of visualizing the tendinous portion of the graft in both sequences. Mean T1rho values gradually decreased during the first year after ACL reconstruction (Fig. 2). Such trends were more prominent in AMB than in PLB. In contrast, reduction of T2 map values was dull in both AMB and PLB during the first year. When temporal changes were individually focused, T1rho values decreased more consistently among patients than T2 map values (Fig. 3).

Mean anteroposterior laxity increased from 0.7 to 1.6 mm during the observation period of 4 years (Table 2). Second-look arthroscopy performed in 18 patients at a mean of 18.9 months indicated a result of “taut” in 13 cases, “mildly lax” in 2 cases, and “lax” in 3 cases for AMB and “taut” in 10 cases, “mildly lax” in 2 cases, and “lax” in 6 cases for PLB. At 4 years postoperatively, mean Lysholm score was 95.9 ± 6.2, and mean Tegner activity level was 6.0 ± 1.1.

**Table 2:** Number of patients followed-up and clinical outcomes at each time point

	Postop. 1 year	Postop. 2 years	Postop. 4 years
Number of patients	20	17	14
KT-values (mm)^a^	0.8 ± 1.7 (0.7 ± 1.8)^b^	0.9 ± 1.7 (1.1 ± 1.7)	1.6 ± 2.7
Rate of positive pivot shift test	5/20	5/17	3/14
Lysholm score	ND^d^	ND	95.9 ± 6.2^a^
Tegner activity scale	ND	ND	6.0 ± 1.1^a^
Arthroscopic analysis of graft tension^c^	AMB: taut, 13; mildly lax, 2; lax, 3 PLB: taut, 10; mildly lax, 2; lax, 6		

^a^Data are expressed as mean ± standard deviation

^b^Data in parenthesis indicates means ± standard deviation of 14 patients who were followed up until 4 years postoperatively

^c^Second-look arthroscopy was performed at 18.9 months on average. Graft tension relative to normal ACL was categorized as taut, mildly lax, or lax, and results of AMB and PLB are expressed separately

^d^*ND* not determined

**Fig. 4:**
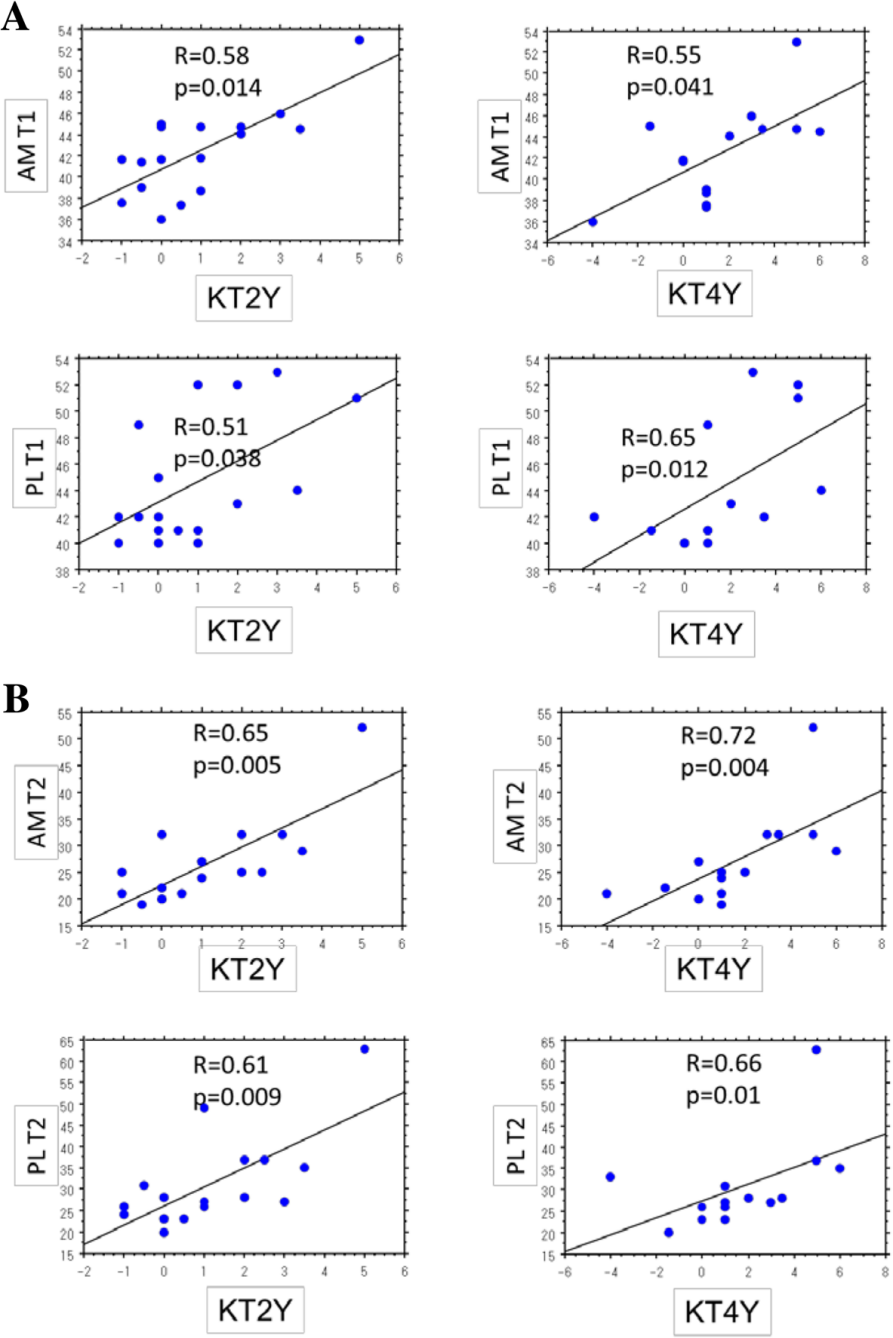
Correlation coefficients and *p* values between T1 rho (**a**) or T2 map values (**b**) and side-to-side differences in anteroposterior laxity as measured with the KT-1000 at 2 and 4 years postoperatively

Correlation analysis revealed that T1rho values of both AMB and PLB at 1 year correlated significantly with anteroposterior laxity at 2 and 4 years (Table 3; Fig. 4). However, neither bundle demonstrated significant correlations between T1rho value and rate of positive pivot shift test at 4 year. At the same time, T2 map values of both AMB and PLB demonstrated a significant correlation with anteroposterior laxity at both 2 and 4 years and pivot shift test results at 4 years. Both T1rho and T2 map values failed to exhibit significant correlations with arthroscopic evaluations of actual graft tension, which was commonly seen for both AMB and PLB.

**Table 3:** Correlation coefficients between T1rho/T2 map values at 1 year and clinical variables

	KT 1 year	KT 2 years	KT 4 years	Pivot shift 4 years	AS grade
T1rho AM	0.39 (0.086)*	0.58 (0.014)^†^	0.55 (0.041)^†^	0.43 (0.057)	− 0.033 (0.89)
T1rho PL	0.042 (0.86)	0.51 (0.038)^†^	0.65 (0.012)^†^	0.41 (0.072)	0.088 (0.73)
T2 AM	0.43 (0.06)	0.65 (0.005)^†^	0.72 (0.004)^†^	0.55 (0.01)^†^	− 0.28 (0.25)
T2 PL	0.31 (0.19)	0.61 (0.009)^†^	0.66 (0.01)^†^	0.68 (0.001)^†^	− 0.30 (0.21)

*Data are expressed as Spearman’s correlation coefficient and *p* value in parentheses

^†^*p* < 0.05 is considered statistically significant

## Discussion

Many studies have evaluated cartilage and meniscus degeneration and injury quantitatively, using T1rho and T2 map sequences [11, 22–24]. These sequences are capable of detecting and mapping the early stages of cartilage degradation [12, 13, 22], but no reports appear to have described the evaluation of ACL graft maturation using these sequences. T1rho value has been reported to increase linearly with decreasing PG content [16, 22]. The breakdown of PG increases the density of mobile protons, resulting in an increased T1rho value [22]. T2 map value reportedly reflects not only water content [1], but also the presence of collagen anisotropy and PG depletion [23]. Measurement of T1rho or T2 map values may have the potential to probe subtle changes in the extracellular matrix of ACL graft in a noninvasive manner.

The present study had two important findings. First, color coding images of both T1rho and T2 map sequences successfully visualized the purely tendinous portion of the ACL graft in the axial plane. T1rho and T2 map values of the tendinous portion gradually decreased in the first operative year. Second, both T1rho and T2 values at 1 year correlated well with anteroposterior knee laxity at 2 and 4 years, suggesting that both sequences could be used as surrogate measures of graft function during the postoperative course of ACL reconstruction. If increased graft signals can be assumed to indicate failed or delayed graft maturation in this study, clinical significance of graft signals can be seen for determining an appropriate timing to return to sport activities on an individual basis. Most surgeons to date have allowed patients to return to the original sport activities according to the time from surgery [25], isokinetic strength of muscles [26], one-leg hop test [27], and so on. No previous studies have used MRI findings as a measure for return to the sport due to a lack of reliability in the MRI assessment of ACL graft maturation.

To date, difficulty in detecting poorly functioning graft by MRI has been reported. One possible reason is the discordance between MRI findings and physical examination of anteroposterior and rotational stability, when using conventional T1- or T2-weighted fast spin echo or proton density-weighted imaging (PDWI) sequences [2, 3]. To the best of our knowledge, only a small number of studies have successfully documented a relationship between actual knee stability and graft intensity on MRI [28–30]. As reported previously, conventional MRI signals include certain degrees of signals from synovial tissues embracing the graft [9], which may be robustly associated with final MRI signals and cause discrepancy between clinical evaluation and MRI findings of the graft. In primary ACL injuries, conventional MRI sequences to date have been shown to offer sufficient diagnostic efficacy, but this is not the case after ACL reconstruction. T1rho and T2 map sequences would fill the gap between MRI findings of the graft and actual knee stability and function.

Hypervascularity of the synovial membrane at 3–6 months may increase mean graft signals in the axial plane, which may obscure signals from the tendinous portion of the graft. This might be the case with the PDWI sequence, which has been perceived as a common sequence for not only cartilage, but also ACL. Although no significant differences were identified, T2 map values of PLB appear to increase from 3 to 6 months postoperatively and decrease thereafter, resembling the temporal pattern of conventional T2, PDWI, and gradient echo T2 sequences reported by previous studies [28, 30, 31]. This may suggest that PLB maturation was abrogated or that segregation of the purely tendinous portion from the surrounding synovium was difficult with the T2 map sequence. Ntoulia et al. assessed temporal changes in graft signals using Gd-DTPA and concluded that synovial tissue embracing the graft continued to display high intensity throughout the course of graft maturation, whereas the volume of synovial tissue peaked at 6 months and decreased until 12 months [9]. The peak graft intensity at 6 months documented in numerous past studies using conventional sequences may be attributable to an increased volume of synovial tissue. Jansson et al. proposed the same insight, that periligamentous tissue surrounding the graft largely contributed to increased graft intensity at 6 months postoperatively [32]. Detection of signals from the purely tendinous portion of the graft is important for proper assessment of graft maturation. In the present analysis with T1rho sequence, ROIs were easily set in the purely tendinous portion of AMB and PLB without color coding images, achieving substantial segregation from surrounding synovial tissue.

Some limitations need to be considered when interpreting the present results. First, as the T1rho sequence is reportedly more sensitive than the T2 map sequence for detecting early cartilage degeneration [33], some differences between sequences were considered to exist when examining ACL graft. However, except for gray-scale visualization and statistical correlations with the pivot shift test result, the two sequences demonstrated broadly similar results. The lower proportion of PG content in ligamentous tissue may favor the T2 map sequence. Second, consistently defining ROIs on purely tendinous portions of graft was difficult, even when using color coding images. A learning curve definitely exists to maintaining the reproducibility of measuring T1 rho and T2 values. Third, one of three cases with possible graft failure (i.e., anterior-posterior laxity ≥4 mm at 4-year postoperatively) exceeded 1 SD of T1rho and T2 map values. The remaining two cases exhibited higher values but still showed a linear correlation. The sample size was insufficient to confirm the feasibility of the two sequences to detect patients with failed graft maturation with adequate statistical power. Moreover, 6 of the 20 patients were lost to follow-up at 4 years postoperatively, because the patients had to undergo both T2-mapping and T1rho sequences of MRI at one session. Such poor follow-up rate substantially decreased a statistical power. Further studies are warranted to determine cutoff values for graft maturation failure using a larger number of cases. These cutoff values are useful as objective surrogate measures of successful graft maturation, at which point new biological interventions would be planned to accelerate graft maturation after ACL reconstruction.

## Conclusions

The present study was the first trial to report the feasibility of T1rho and T2 map sequences in objectively monitoring the course of graft maturation after ACL reconstruction. Both sequences successfully visualized the purely tendinous portion of the ACL graft, and values decreased with increasing graft maturation during 1 year postoperatively. Both T1rho and T2 map values at 1 year correlated significantly with anteroposterior laxity of the knee joint at 4 years, indicating that both sequences are useful to evaluate graft function and possible timing of the return to sport activities.

## Abbreviations

ACL: Anterior cruciate ligament
AMB: Anteromedial bundle
MCL: Medial collateral ligament
MRI: Magnetic resonance imaging
PCL: Posterior cruciate ligament
PLB: Posterolateral bundle
